# Niches and Genotypes Determine the Diversity and Composition of Microbiomes After Herbicide Treatment in *Beckmannia syzigachne*

**DOI:** 10.3390/plants14060876

**Published:** 2025-03-11

**Authors:** Kehan Bai, Yulan Ouyang, Jiale Qi, You Zhan, Junzhi Wang

**Affiliations:** 1 Hunan Institute of Plant Protection, Hunan Academy of Agricultural Sciences, Changsha 410125, China; kehanb123@163.com; 2 College of Plant Protection, Hunan Agricultural University, Changsha 410128, China; oyyl0717@163.com (Y.O.); qjl@stu.hunau.edu.cn (J.Q.); zhany@stu.hunau.edu.cn (Y.Z.)

**Keywords:** *Beckmannia syzigachne*, root niches, bacteria, mesosulfuron-methyl, endophytic fungi

## Abstract

Plant-associated microbes play a crucial role in plant adaptability by facilitating nutrient acquisition, growth, and stress resistance. However, the effects of herbicides on microbial communities in different root-associated niches and their impact on weed–microbe interactions are not well understood. *Beckmannia syzigachne*, a problematic weed, reduces crop yield and quality. In this study, we investigated bacterial and fungal community diversity in *B. syzigachne* using 16S and internal transcribed spacer (ITS) rRNA sequencing. Significant differences were observed in bacterial community structure across four root-associated niches, with diversity decreasing from bulk soil to endosphere. The sensitive genotype exhibited higher bacterial diversity compared to the resistant biotype, indicating that sample type is the primary factor influencing microbial community composition, with genotype playing a secondary role. Additionally, we examined fungal communities in sensitive and resistant populations, identifying 271 fungal operational taxonomic units (OTUs). *Ascomycota*, *Basidiomycota*, and *Rozellomycota* were dominant in the sensitive population, while the resistant population contained two unique OTUs, *Saccharomyces* sp. and *Apiotrichum montevideense*, which were absent in the sensitive population. This study provides insights into how bacterial and fungal communities in *B. syzigachne* populations respond to herbicide exposure, contributing to a deeper understanding of weed–microbe interactions.

## 1. Introduction

*Beckmannia syzigachne* is a diploid grass weed that infests agricultural fields, leading to significant reductions in crop productivity [1,2]. Herbicides targeting acetyl-CoA carboxylase (ACCase) and acetolactate synthase (ALS) are primary tools for *B. syzigachne* control. However, prolonged use of ACCase inhibitors over two decades has led to widespread resistance in this weed, attributed predominantly to target-site mutations in the *ACCase* gene [3,4,5,6]. Herbicide resistance in weeds typically arises through two mechanisms: target-site resistance (TSR), which involves structural alterations in herbicide-binding enzymes, and non-target-site resistance (NTSR), which involves enhanced metabolic detoxification or antioxidant responses [7,8]. For instance, *BsCYP81Q32* overexpression produces metabolic resistance to mesosulfuron-methyl in *B.syzigachne* [6]. Asiatic dayflower (*Commelina communis*) populations resistant to atrazine exhibit no *psbA* gene mutations but instead rely on elevated antioxidant enzyme activities to mitigate herbicide-induced oxidative stress [9]. This highlights the need to explore both TSR and NTSR mechanisms in evolving resistance management strategies.

To address ACCase resistance, ALS inhibitors such as mesosulfuron-methyl (a sulfonylurea herbicide) have been widely adopted due to their high efficacy and low environmental toxicity [10,11,12]. Mesosulfuron-methyl degrades through photolysis on leaf surfaces, microbial metabolism in soil, and plant metabolic processes [13]. The use of herbicides can enhance crop yields, but herbicide residues have led to a series of environmental issues [14]. Bending et al. demonstrated that herbicide residues in soil can affect microbial activity and diversity [15]. However, there are few reports on the long-term degradation, accumulation, and ecological effects of mesosulfuron-methyl residues. Therefore, understanding the ecological impact of mesosulfuron-methyl is crucial when assessing its potential environmental risks. Microorganisms play a vital role in the degradation of pesticide residues in the environment, and bioremediation has garnered significant attention [16,17]. The microbial degradation of sulfonylurea herbicides is a primary method for soil detoxification [18]. Soil, as a vast reservoir of diverse microorganisms, harbors many species adapted to harsh environments, capable of degrading organic pollutants [19]. Therefore, further research is needed to explore the degradation behavior of mesosulfuron-methyl in soil and its intrinsic relationship with microorganisms.

Numerous studies have confirmed that plants selectively recruit special microbes from soil to assemble characteristic but very complex microbial communities to resist environmental stress. Endophytes live in plant tissues without expressing symptoms or visible signs [20,21,22,23,24,25,26]. Plant endophytes can promote the growth and development of host plants and improve the ability of plants to resist abiotic stress [27]. Endophytes of *Sphingomonas* sp. enhance *Polypogon fugax* resistance to quizalofop-*p*-ethyl [28]. The dynamics and structure–function relationships of *B. syzigachne* microbial communities have not been studied.

This study investigates the ecological interplay between mesosulfuron-methyl exposure, *B. syzigachne* biotypes, and associated microbial communities. Our objectives are twofold: (1) to compare microbial composition across root-soil niches, and (2) to analyze microbiome diversity in relation to plant genotypes. By integrating herbicide resistance mechanisms with microbial ecology, this work aims to inform sustainable weed management practices.

## 2. Materials and Methods

### 2.1. Experimental Design for B. syzigachne Growth and Sampling

Soil for pot experiments was collected from a rapeseed field in Changsha, China. The soil was air-dried, sieved through a 2 mm mesh, and thoroughly homogenized. Physiological and biochemical parameters were measured, including pH 8.36, total nitrogen content (1.63 g kg^−1^), and total organic carbon content (0.89%). S and R populations were treated at the recommended dose of 11.25 g ai. ha^−1^ in the field to compare the effects on aboveground parts and root growth. To simulate a post-herbicide application environment, mesosulfuron-methyl (Stellar Anon Biotechnology Co., Ltd., Qingdao, China) was applied at a dose of 1.5 g ai. ha^−1^ at 3–4 leaves stage in *B. syzigachne*. Two populations of *B. syzigachne* with differing sensitivities to mesosulfuron-methyl (S and R) were selected. Seeds were sterilized and germinated under sterile conditions. The germinated seedlings were then transplanted into pots (9 cm diameter × 10 cm height) containing 0.3 kg of prepared soil. The pots were placed in a glasshouse under natural light and maintained at room temperature. Sterile water was used for irrigation to maintain the soil moisture content at 60% to 70% of field capacity. All pots received the same nutritional supplementation. After 21 days of cultivation, samples were collected to record the fresh weight of both the above-ground and root parts of the plants. Each treatment had five biological replicates, with six seedlings per pot. Pots without plants served as bulk soil controls.

### 2.2. Comparison of Root Niches Bacterial in the S and R Populations

#### 2.2.1. Sample Preparation and DNA Extraction

After 21 days of growth, samples were collected, and fresh weights were recorded. Samples from various root-associated environments, including bulk soil, rhizosphere soil, rhizoplane, and root tissues, were collected for 16S rRNA high-throughput sequencing. Bulk soil: collected from pots without plants. Rhizosphere soil: soil adhering to roots was gently shaken off and manually removed from the root surface. Approximately 2 mm of soil directly adjacent to the roots was collected. Rhizoplane soil: after removing rhizosphere soil, roots were placed in sterile PBS and subjected to ultrasonic treatment at 50–60 Hz for 30 s to remove adhering soil particles. The resulting suspension was considered rhizoplane soil. Root Tissues: Clean root surfaces were treated with 75% ethanol for 1 min, followed by five rinses with sterile water to prepare root samples for internal microbial analysis. All root-associated samples from the different ecological niches were stored at −80 °C. DNA extraction was performed according to standardized protocols suitable for 16S rRNA sequencing to analyze the microbial communities associated with the root environments.

#### 2.2.2. 16S rRNA Amplifying and Illumina MiSeq Sequencing

Genomic DNA was extracted from various samples, including bulk soil, rhizosphere soil, rhizoplane soil, and root tissues, using standardized protocols optimized for soil and plant materials to ensure high-quality DNA suitable for downstream applications. The V3–V4 region of the bacterial 16S rRNA gene was amplified using the forward primer 338F and reverse primer 806R (Table S1). The PCR mixture contained: 5 μL of 5× buffer, 0.25 μL of Fast Pfu DNA Polymerase (5 U/μL), 2 μL of dNTPs (2.5 mM), 1 μL of each primer (10 μM), 1 μL of DNA template, and 14.75 μL of ddH_2_O. Following amplification, PCR products were purified and quantified. The amplicons from each sample were then combined in equal proportions for sequencing.

Sequencing was conducted on the Illumina MiSeq platform using the MiSeq Reagent Kit v3 at Shanghai Personal Biotechnology Co., Ltd. (Shanghai, China). Libraries were prepared with the Illumina TruSeq Nano DNA LT Library Prep Kit. The quality of the library was assessed using a 1 μL aliquot with the Agilent High Sensitivity DNA Kit on an Agilent Bioanalyzer (Agilent Technologies, Santa Clara, CA, USA). Microbiome bioinformatics analysis was performed using QIIME2 2019.4 [29], which included quality control, sequence alignment, and taxonomic classification to analyze the microbial communities across different root environments.

#### 2.2.3. Bioinformatics Analysis

Sequence data analysis was conducted using QIIME and R packages (v3.2.0). Alpha diversity metrics at the ASV (amplicon sequence variant) level were calculated, including the Chao1 richness estimator, Observed Species, Shannon diversity index, Simpson index, Faith’s Phylogenetic Diversity (PD), Pielou’s evenness, and Good’s coverage, based on the ASV table in QIIME2. These metrics were visualized using box plots to compare ASV richness and evenness among samples. Ranked abundance curves at the ASV level were generated to evaluate the distribution of ASVs within each sample, offering insights into the richness and evenness across different samples. To investigate structural differences in microbial communities among samples, beta diversity analysis was performed using various distance metrics. Specifically, the Jaccard metric was employed to measure community similarity based on the presence/absence of species [30]. These analyses were essential for identifying the diversity and composition of microbial communities within the samples and for assessing the impact of factors such as genotype and environment on microbial community structure. Microbial functions were inferred using PICRUSt2 (Phylogenetic Investigation of Communities by Reconstruction of Unobserved States), with the MetaCyc and KEGG databases serving as references.

### 2.3. Comparison of Leaf Endophyte Fungi in the S and R Populations

In studies on weed–microbe interactions, the role of endophytes in weeds is often overlooked. To investigate the relationship between endophytic microorganisms and sensitive weeds, leaf samples were collected from both resistant (R) and sensitive (S) populations at the four- to five-leaf stage, with five replicates per population, totaling 10 samples (S1–S5, R1–R5). To eliminate epiphytic fungi, leaf fragments underwent a sterilization protocol: first rinsed with sterile water, then soaked in 70% ethanol for 2 min, followed by treatment with 2.5% sodium hypochlorite for 5 min, and finally washed five times with sterile water. The sterilized leaves were dried using sterile absorbent paper [31], cut into 2–3 cm fragments with a sterile scalpel, and stored at −80 °C under sterile conditions.

Total microbial genomic DNA was extracted from the leaf samples. The ITS2 region of fungal rRNA genes was amplified employing the primer pairs ITS1F and ITS2R (Table S1). The purified amplicons were combined in equimolar concentrations and sequenced using the Illumina MiSeq PE300 platform (Illumina, San Diego, CA, USA), following the standard procedures provided by Majorbio Bio-Pharm Technology Co., Ltd. (Shanghai, China). The optimized sequences were then grouped into operational taxonomic units (OTUs) via UPARSE 7.1 [32,33] with a 97% sequence similarity threshold. To reduce the impact of sequencing depth on alpha and beta diversity assessments, the most frequent sequence within each OTU was chosen as the representative sequence.

Statistical analyses were conducted in R 3.5.1. The raw FASTQ files were de-multiplexed with a custom Perl script and subsequently quality-filtered using fastp version 0.19.6 [34] and merged by FLASH version 1.2.7 [35]. Using the OTU data, rarefaction curves along with alpha diversity metrics, such as observed OTUs, Chao1 richness, Shannon index, and Good’s coverage, were computed with Mothur version 1.30.1 [36]. To assess the similarity among microbial communities across different samples, principal coordinate analysis (PCoA) was performed using Bray–Curtis dissimilarity, implemented with the Vegan v2.5-3 package.

### 2.4. Statistical Analyses

To compare the relative abundance of microbial taxa and α-diversity indices among different genotypes (populations) and ecological niches, a one-way analysis of variance (ANOVA) was performed, followed by Tukey’s Honest Significant Difference (HSD) test (*p* < 0.05).

## 3. Results

### 3.1. Mesosulfuron-Methyl Treatment Reduces Biomass in Sensitive B. syzigachne Populations

**Figure 1:**
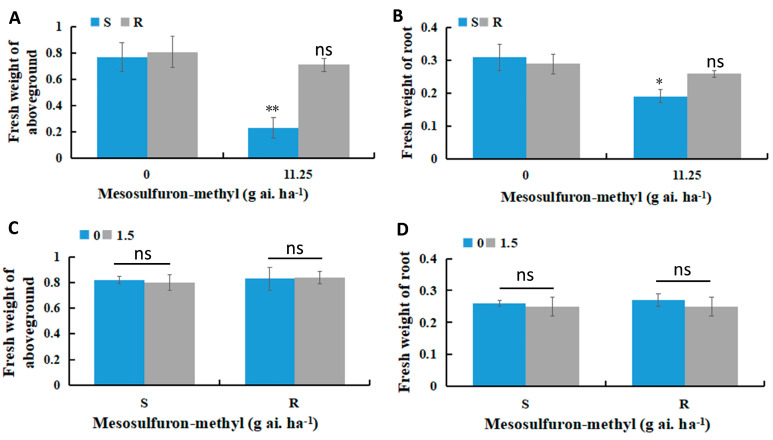
(**A**) Fresh weight of aboveground of susceptible (S) and resistant (R) populations at 11.25 g ai. ha^−1^ mesosulfuron-methyl; (**B**) Fresh weight of root of S and R populations at 11.25 g ai. ha^−1^ mesosulfuron-methyl; (**C**) Fresh weight of the aboveground of S and R populations at the dose of 1.5 g ai. ha^−1^ mesosulfuron-methyl; (**D**) Fresh weight of the root of S and R populations at the dose of 1.5 g ai. ha^−1^ mesosulfuron-methyl. *, *p* < 0.05. **, *p* < 0.01. ns, *p* > 0.05.

Under a mesosulfuron-methyl treatment at a dose of 11.25 g ai. ha^−1^, the biomass of the S population was reduced compared to the untreated control, while the aboveground biomass of the R population remained unaffected (Figure 1A). Additionally, the fresh weight of the roots in the S population significantly decreased (Figure 1B). In pot experiments conducted under herbicide stress (1.5 g ai. ha^−1^ mesosulfuron-methyl) for 21 days, the aboveground fresh weight of the S population was not significantly different from that of the R population. However, the aboveground fresh weight of the S population was reduced by 2.4% compared to the R population (Figure 1B). Similarly, the root fresh weight of the S population was reduced by 3.8% (Figure 1C,D). The observed trend in root fresh weight paralleled that of the aboveground biomass, though it did not impact the overall growth of *B. syzigachne*.

### 3.2. Niche and Genotype Influence the Diversity and Composition of B. syzigachne Root Microbiomes

To assess the impact of niche and genotype on the microbial communities of *B. syzigachne*, we analyzed bacterial communities from four distinct niches: bulk soil, rhizosphere soil, rhizoplane, and root endosphere. This study focused on two genotypes of *B. syzigachne* (sensitive, S, and resistant, R populations) using the V3–V4 region of the 16S rRNA gene. After filtering out chimeric and organellar sequences, a total of 2,085,256 V3–V4 sequences from 35 samples were analyzed. These sequences were classified into 393 bacterial operational taxonomic units (OTUs) based on a 97% similarity threshold (Figure S1A). The sequence lengths were predominantly between 350 and 400 base pairs (Figure S1B).

To analyze the bacterial species composition across different niches of *B. syzigachne* roots, we performed statistical analyses on the rarefied ASV/OTU tables. This provided detailed compositions of the microbial communities at various taxonomic levels for each sample (Table S2, Figure S1C). Marked divergence were observed in bacterial communities across different niches of the root system. At the genus level, the species composition of bulk soil, rhizosphere soil, and rhizoplane showed significantly higher species abundance compared to the root endosphere. Among the top 20 genera, *Bathyarchaeia* exhibited the highest abundance (Figure S1D). The community taxonomic composition was interactively visualized using Krona [37]. At the phylum level, the most dominant groups were Proteobacteria (51%), Bacteroidetes (11%), Firmicutes (11%), Actinobacteria (9%), Acidobacteria (5%), Chloroflexi (4%), Planctomycetes (2%), and Gemmatimonadetes (2%) (Figure S2).

**Figure 2:**
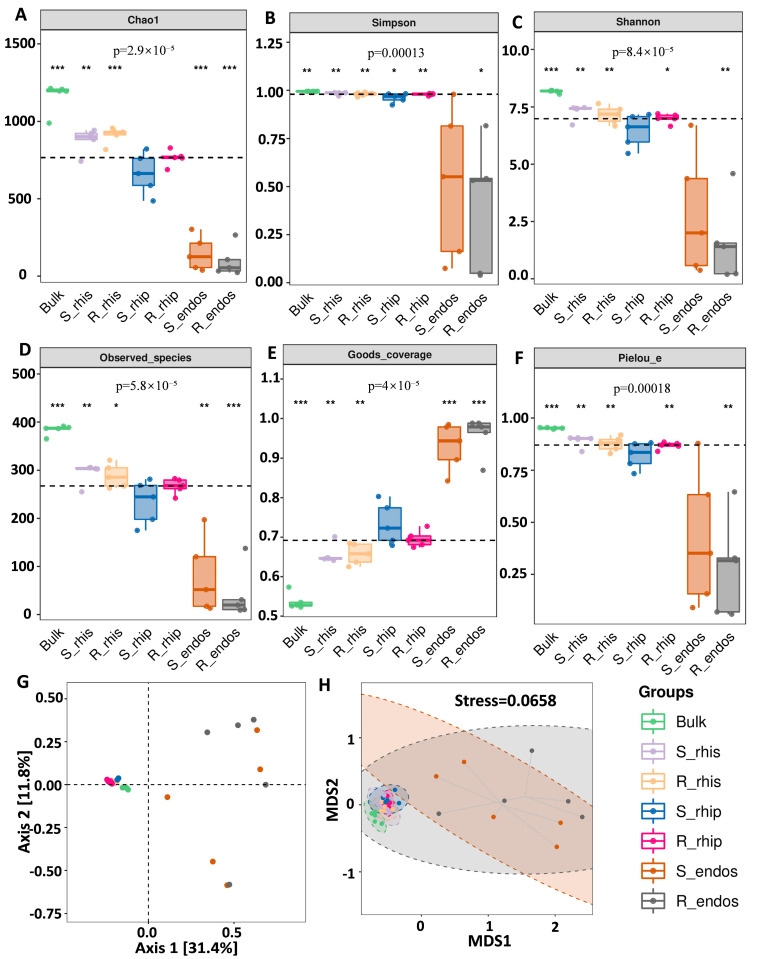
Diversity and composition of *B. syzigachne* microbiomes across four niches and two genotypes. Alpha diversity indices: (**A**) Chao1; (**B**) Simpson; (**C**) Shannon; (**D**) Observed species; (**E**) Goods coverage; (**F**) Pielou’s evenness. Each panel corresponds to an alpha diversity index, indicated in the gray area at the top. In each panel, the horizontal axis represents the group labels, and the vertical axis represents the corresponding alpha diversity index values. (**G**) PCoA analysis of sample two-dimensional ordination plot, where each point represents a sample, and different colored points indicate different samples. (**H**) NMDS two-dimensional ordination plot, where each point represents a sample, and different colored points indicate different samples. The horizontal axis is the grouping label and the vertical axis is the value of the corresponding alpha diversity index. In the box diagram, the meanings of symbols are as follows: upper and lower end lines of the box, upper, and lower quartile range (IQR); Median line, median; Top and bottom edges, Max and min inner circumference values (1.5 times IQR). *, *p* < 0.05. **, *p* < 0.01. ***, *p* < 0.001.

Bacterial α-diversity, represented by six diversity indices (Chao1, Shannon, Simpson, Pielou’s evenness, observed species, and Good’s coverage), showed significant differences among the different niches (Figure 2A,F). Specifically, bacterial α-diversity was highest in bulk soil and lowest in the root endosphere. From a population perspective, bacterial α-diversity was higher in the S genotype compared to the R genotype. Within each tissue type, there were differences in bacterial α-diversity between the two *B. syzigachne* populations. The species accumulation curves (Specaccum) plotted from the ASV/OTU abundance matrix for each sample indicated that the 35 samples were sufficient to estimate the richness of the bacterial communities (Figure S3A). These results suggest that the variations in root-associated bacterial communities are primarily driven by the specific root-associated niches.

To explore the differences in microbial community composition (beta diversity), PCA analysis based on the OTU/ASV table demonstrated clear spatial separation of microbial community structures across different niches, indicating marked divergence in bacterial community composition among these niches (Figure 2G). Additionally, NMDS two-dimensional ordination plots based on Jaccard and Bray–Curtis distances illustrated the distribution of samples from different niches (Figure 2H). This further confirmed the distinct community structures among root-associated niches, highlighting that the root endosphere harbors bacterial communities that are markedly different from those in other niches.

### 3.3. Genotypes Differentiation Analysis in Different Niches of B. syzigachne Roots

**Figure 3:**
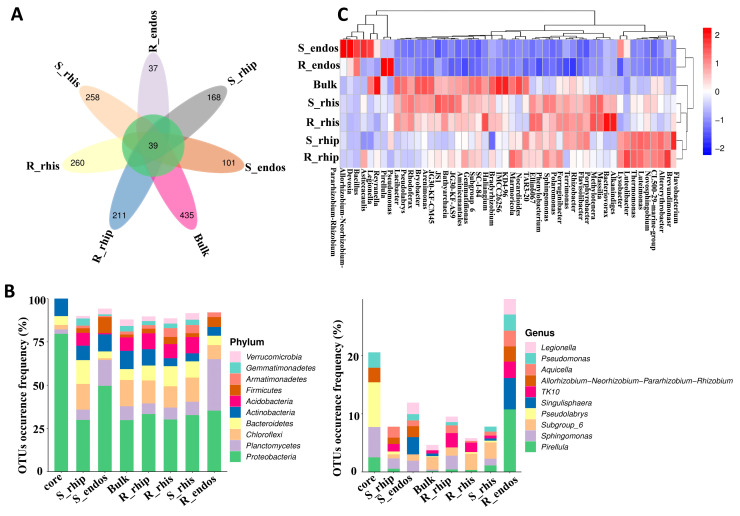
(**A**) Petal Diagram for Sample ASV/OTU: Each oval in the diagram represents an individual sample. The overlapping area in the center of all ovals indicates the ASVs/OTUs shared by all samples. (**B**) Bar chart of ASV/OTU Counts for Different Petal Diagram Regions: The horizontal axis displays ASV/OTU sets associated with various regions of the petal diagram. (**C**) Heatmap of Species Composition at the Genus Level.

To investigate the compositional differences in bacterial communities in various root niches, we used ASV/OTU abundance tables to create a Venn diagram (Figure 3A). A total of 39 OTUs were shared among Bulk soil, S-rhizosphere, R-rhizosphere, S-rhizoplane, R-rhizoplane, S-endosphere, and R-endosphere samples. Bulk soil exhibited the highest number of unique OTUs (435), followed by R-rhizosphere (260), S-rhizosphere (258), R-rhizoplane (211), S-rhizoplane (168), S-endosphere (101), and R-endosphere (37).

We further analyzed the taxonomic distribution of species within each niche using petal diagrams. At the phylum level, the abundance of ASVs/OTUs in each niche was quantified. Proteobacteria dominated all samples (except R-endosphere) with relative abundances greater than 25% (Figure S3B). At the genus level, the most dominant genera included *Legionella*, *Pseudomonas*, *Aquicella*, *Allorhizobium*, *TK10*, *Singulisphaera*, *Pseudolabrys*, *Subgroup_6*, *Sphingomonas*, and *Pirellula* (Figure 3B). To further compare species composition differences among samples, a heatmap was generated using the abundance data of the top 50 genera (Figure 3C). The genus *Reyranella* was significantly enriched only in Bulk soil samples. In S-endosphere and R-endosphere samples, *Pirellula* and *Pseudomonas* showed higher abundance in R-endosphere than in S-endosphere. In S-rhizosphere and R-rhizosphere samples, *Alkanindiges*, *Bacteriovorax*, and *Haliangium* were more abundant in R-rhizosphere compared to S-rhizosphere. *Asticcacaulis* was more abundant in R-rhizoplane than in S-rhizoplane.

Finally, Orthogonal Projections to Latent Structures Discriminant Analysis (OPLS-DA) was employed to illustrate the differences in species abundance compositions among the four niches of *B. syzigachne* roots (Figure S4). This analysis revealed significant variations in the microbial communities among the different root-associated niches.

### 3.4. Potential Sources and Co-Occurrence Network of Root-Associated Microbiome

To investigate the root-associated microbial communities in resistant and susceptible *B. syzigachne* populations under exposure to mesosulfuron-methyl, we conducted an association network analysis. This analysis utilized Zi (within-module connectivity) and Pi (among-module connectivity) scores to identify key species. Using these scores, the nodes (ASV/OTU) in the network were categorized into four groups: peripherals, connectors, module hubs, and network hubs (Figure S5A).

**Figure 4:**
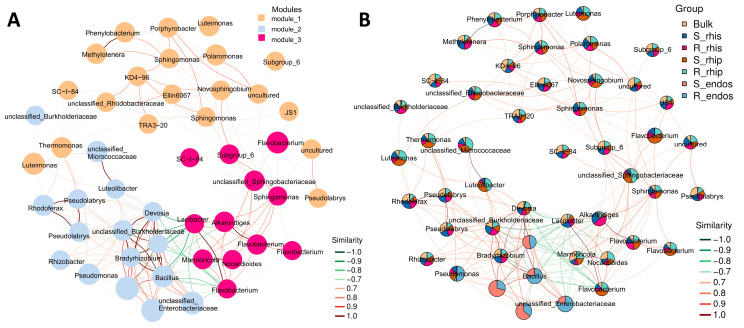
(**A**) Modular sub-network of dominant species: Nodes represent ASVs or OTUs in the samples, with node size proportional to their abundance (measured in log2(CPM/n)). Different colors indicate the modules with the highest number of nodes, up to a maximum of 10 nodes per module. (**B**) Sub-network of dominant species with group abundance pie charts: Nodes represent ASVs or OTUs in the samples, with node size proportional to their abundance (measured in log2(CPM/n)). The sub-network shows only the top 50 ASVs/OTUs by average abundance across samples. Edges represent correlations between connected nodes, with red lines representing positive correlations and green lines denoting negative correlations.

At the phylum level, the dominant groups included Proteobacteria, Bacteroidetes, Actinobacteria, Acidobacteria, Chloroflexi, Firmicutes, Gemmatimonadetes, Verrucomicrobia, Planctomycetes, and Crenarchaeota, Network nodes were distributed across 10 bacterial phyla (Figure S5B). When modularizing the network nodes, they were divided into three major modules (Figure 4A). Proteobacteria, Bacteroidetes, Actinobacteria, Acidobacteria, and Chloroflexi were predominant in modules 1, 2, and 3. Additionally, we analyzed the subnetwork graphs of dominant species’ abundance pie charts in different niches (Bulk soil, S-rhizosphere, R-rhizosphere, S-rhizoplane, R-rhizoplane, S-endosphere, and R-endosphere) (Figure 4B). This research provides valuable insights into the complex interactions within microbial communities and their responses to environmental changes.

### 3.5. Functional Potential Prediction

The functional potential of microbial communities in the niches of *B. syzigachne* was predicted using PICRUSt2. This tool analyzes 16S rRNA gene sequences to infer the gene functional profiles of the samples, focusing on four different niches: bulk soil, rhizosphere soil, root surface, and endosphere. The majority of predicted functions were related to metabolic pathways, including amino acid metabolism, carbohydrate metabolism, and the metabolism of cofactors and vitamins. The endosphere niche exhibited the highest abundance of genes associated with carbohydrate metabolism, likely linked to root exudates serving as a carbon source for microbes. Genes related to nitrogen metabolism were also prevalent, particularly in the root surface and rhizosphere soil, indicating potential nitrogen cycling activity in these niches (Figure S6). These findings highlight the significant functional roles of root-associated microbial communities in nutrient cycling and metabolism within different root-related environments.

### 3.6. Endophytic Fungal Microbiota in S and R Populations

**Figure 5:**
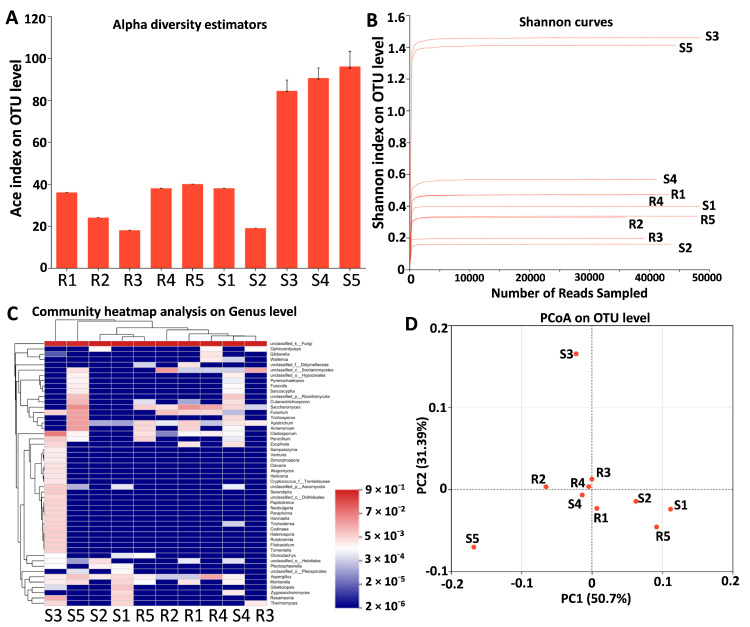
Alpha diversity estimators and beta diversity. (**A**) Alpha diversity estimators in the S and R populations. (**B**) The x-coordinate represents the amount of randomly extracted sequencing data; the ordinate and the Shannon index. (**C**) Community heatmap analysis at the genus level in the S and R populations. (**D**) PCoA at the OTU level in the S and R populations.

Illumina sequencing generated 423,980 fungal sequences, with an average length of 296 bp after raw reads were filtered and qualified. In total, 271 fungal OTUs associated with *B. syzigachne* were identified across 8 phyla, 25 classes, 53 orders, 95 families, 139 genera, and 184 species (Table S3). Alpha diversity indices derived from fungal OTUs of S and R populations revealed that R populations harbored fewer diverse fungal OTUs compared to S populations (Figure 5A). In total, 225,875 and 207,105 raw reads were obtained from the amplicon libraries of fungi in the S and R populations, respectively, and the average read lengths before processing were 294 bp and 299 bp (Table S3). We assessed the coamplification of ITS regions (ITS1F and ITS2R) and examined the number of reads that could not be clearly classified at the phylum level (Table S3). Rarefaction curves were generated, illustrating the relationship between the number of OTUs and Shannon index with the total sequence count (Figure 5B). To compare the fungal community composition and assess the differences between the S and R populations, beta diversity was evaluated at the genus level for fungi (Figure 5C), and higher fungal diversity measures were observed in the S population than in the R population. Overall similarities in fungal community structures among samples were displayed using principal coordinates analysis (PCoA) (Figure 5D). These findings indicate that the fungal diversity of S was higher than that of the R population.

**Figure 6:**
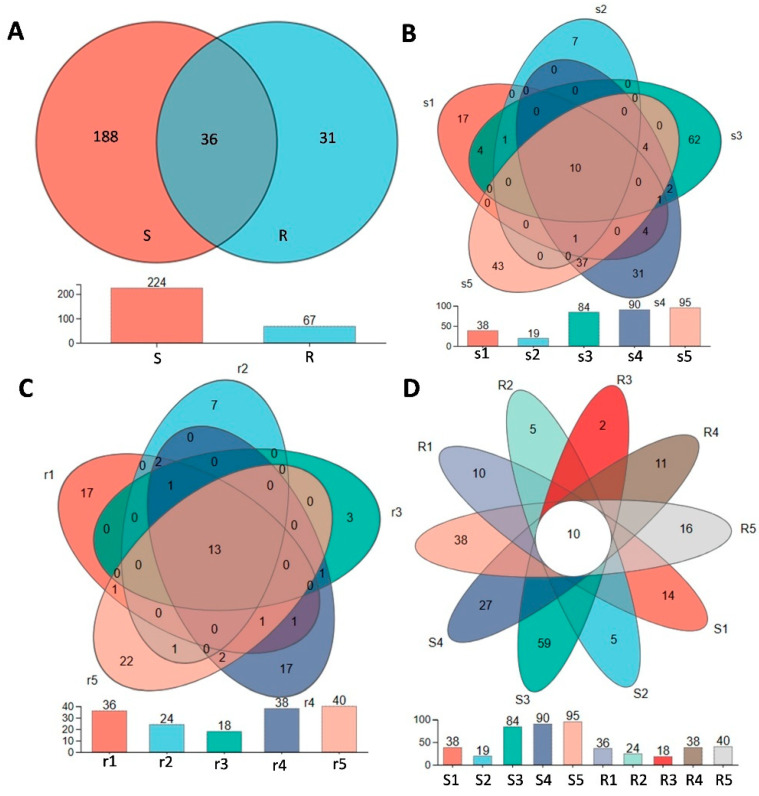
Venn diagrams depicting fungal operational taxonomic units (OTUs) in *B. syzigachne* leaf samples, illustrating the number of shared and unique OTUs. (**A**) Number of OTUs in the S and R populations. (**B**) Number of OTUs in the S population. (**C**) Number of OTUs in the R population. (**D**) Comparison and analysis of the number of OTUs in the S and R populations. Different circles represent various plant sample types, with their intersections indicating the fungal OTUs common to those samples.

For the fungal community, a total of 432,444 validated sequences were obtained after passing quality filters. The number of OTUs was higher in the S population than in the R population (Figure 6A). At the species level of *B. syzigachne* fungi, there were 154 species of fungi in the S population, while there were only 43 species of fungi in the R population, and 26 species were shared by the S and R populations (Figure S7a). The distribution proportions of dominant species within each sample (or group) and across different samples (or groups) were illustrated using a visualization circle diagram (Figure S7b). *Ascomycota*, *Basidiomycota*, and *Rozia* were the dominant phyla in the S population. By further analyzing the diversity of endophytic fungi in the S and R populations, it was found that the five biological replicates in the S population had 10 common OTUs (Figure 6B); they were OTU17, OTU98, OTU95, OTU96, OTU92, OTU84, OTU87, OTU86, OTU89, and OTU88. The five biological replicates of the R population had a total of 13 OTUs (Figure 6C); they were OTU17, OTU98, OTU96, OTU92, OTU95, OTU25, OTU21, OTU83, OTU87, OTU86, OTU84, OTU89, and OTU88. Among them, the ten OTUs shared by the five S samples were also shared by the five biological replicates of R (Figure 6D). However, the R population had two OTUs that were not found in the S population: OTU21 (*Saccharomyces * sp.), and OTU83 (*Apiotrichum montevideense*). Studies have shown that glyphosate is broken down into phosphate ions, carbon dioxide, and water by the enzymes of *Saccharomyces* sp. [38]. *Apiotrichum* also have metabolic and phenotypic plasticity [39]. These results imply that *Saccharomyces* sp. and *Apiotrichum montevideense* may contribute to the resistance of the R population to ALS herbicides.

## 4. Discussion

The ability of microbial communities to adapt to environmental factors, such as soil type, anoxic/oxic conditions, salinity, nutrient availability, and plant community composition, is a key determinant in shaping bacterial community structure [40,41]. In the context of *B. syzigachne* and its response to mesosulfuron-methyl pollution, understanding the microbial composition and its relationship to herbicide resistance is vital for microbial ecology and ecological remediation strategies. However, the complex and dynamic interactions between environmental factors and microbial communities pose challenges for their detailed characterization. The impact of mesosulfuron-methyl on microbial communities and the potential implications for herbicide resistance in *B. syzigachne* provide valuable insights into the ecological and physiological mechanisms underlying this phenomenon.

In this study, we analyzed the impact of different biotypes and niches of *B. syzigachne* on bacterial and fungal community structures. Furthermore, since microorganisms are essential in breaking down residual pesticides in the environment, pollution remediation has received considerable attention [42,43]. Therefore, understanding the impact of mesosulfuron-methyl exposure at the microbial community level is useful for the remediation of mesosulfuron-methyl residues. The application of mesosulfuron-methyl, an ALS inhibitor, had a differential impact on the biomass of S and R populations (Figure 1). While the S population exhibited a significant reduction in biomass upon herbicide treatment (12 g ai. ha^−1^), the R population showed no such decline, suggesting that the resistant individuals possess specific mechanisms to cope with the herbicidal stress. These findings imply that the R population may have evolved metabolic or physiological adaptations, which enable them to either degrade or tolerate the herbicide more effectively than their susceptible counterparts.

It is well established that plants can recruit microbial communities to help mitigate stressors, including herbicide exposure. Root exudates, which increase in response to various stress factors, can enhance microbial diversity and biomass in the rhizosphere, further suggesting a symbiotic relationship between plants and microbes under stress [44]. In this study, we observed significant differences in microbial diversity across different root-associated niches (Figure 2), with the S population supporting higher microbial diversity compared to the R population. This could indicate that the herbicide susceptibility of the S genotype is tied to its microbial community, which is more vulnerable to disruption by mesosulfuron-methyl. Conversely, the R population may harbor microbial communities that are better equipped to withstand herbicide-induced stress, potentially contributing to their overall resistance. Our observation of declining microbial diversity from bulk soil to root endosphere aligns with the rhizosphere filtering model, wherein plants selectively recruit microbes across niches through root exudate-mediated selection. This observation underscores the need to further investigate the specific microbial taxa and functional traits that contribute to herbicide resistance in *B. syzigachne*. While this study provides critical insights into niche-specific microbiome shifts in *B. syzigachne* under herbicide stress, we acknowledge that its snapshot design—sampling microbiomes 21 days post-herbicide application—may overlook dynamic microbial responses during earlier resistance initiation or long-term adaptation. Our functional potential predictions using PICRUSt2 support this hypothesis, revealing distinct functional profiles across different rhizosphere niches, suggesting that microbial communities are specialized to adapt to the unique nutrient conditions of each niche. This highlights the importance of niche-specific functions in microbial resilience to environmental stressors, including herbicide exposure.

Endophytes are traditionally described as fungi that live within plant tissues and either promote plant growth or exist in a commensal relationship with the host [45]. However, the endophytic microbiota of *B. syzigachne* has yet to be comprehensively characterized. Our Illumina sequencing data reveal that the leaf endophytic fungal diversity is higher in the S population than in the R population, which further suggests that microbial community composition could influence herbicide resistance. Notably, endemic yeast species such as *Saccharomyces* sp. and *Apiotrichum montevideense* were found exclusively in the R population. These fungi have demonstrated remarkable metabolic versatility and are known to degrade various pollutants, including glyphosate and phenolic compounds [16,39]. *Apiotrichum montevideense* exhibited activity for esterase-lipase (C8), lipase (C14), valine arylamidase, naphthol-AS-BI phosphohydrolase, α-galactosidase, and β-glucosidase [46]. *Apiotrichum* members have metabolic and phenotypic plasticity [40]. *Apiotrichum montevideense* can oxidize and crack biphenyl, dibenzofuran, and diphenyl ether [47]. The unique enrichment of *Saccharomyces* sp. and *Apiotrichum montevideense* in resistant *B. syzigachne* biotypes positions these fungi as potential microbial biomarkers for field-level resistance monitoring. Their rapid detection via qPCR or metabarcoding could enable early identification of herbicide-resistance hotspots, informing precision herbicide rotation or microbiome-modulating interventions to delay resistance evolution in agroecosystems. The spread of local endophytes and their close relationship with host plants should be beneficial to plant growth and stress resistance. However, the variability in fungal community reproducibility (Figure 5D) likely stems from biological heterogeneity and technical limits. Such stochasticity aligns with plant microbiome studies under stress, emphasizing the need for robust replication and cultivation-based validation to distinguish ecological signals from noise. These results suggest that the endophytic fungi *Saccharomyces* sp. and *Apiotrichum montevideense* may play a role in conferring herbicide resistance in the resistant population. While our study delineates microbiome-herbicide resistance linkages, the absence of metabolite profiling limits mechanistic insights into plant–microbe crosstalk. Further research is needed to elucidate the precise mechanisms by which endophytic fungi confer resistance to herbicides.

The interaction between bacterial and fungal endophytes and their collective impact on plant health and stress resilience remains an underexplored area. The microbial communities in the rhizosphere and internal plant tissues are likely to influence herbicide resistance through a combination of direct interactions with herbicide residues and indirect effects on plant growth and metabolic processes. Investigating these interactions will be crucial for understanding the complex relationship between *B. syzigachne* and its microbial symbionts. Ultimately, a deeper understanding of microbial community dynamics in herbicide-resistant populations could offer novel strategies for managing resistance, potentially by altering microbial compositions to favor herbicide-susceptible communities or by engineering microbial consortia that enhance herbicide degradation.

## 5. Conclusions

This study highlights the distinct bacterial and fungal community compositions across different niches and biotypes of *B. syzigachne* under mesosulfuron-methyl treatment. Root-associated bacterial diversity varied significantly, with α-diversity highest in bulk soil and lowest in the root endosphere. Functional predictions indicated niche-specific microbial specialization in response to environmental conditions. The S population exhibited greater endophytic fungal diversity than the R population, with specific OTUs like *Saccharomyces* sp. and *Apiotrichum montevideense* exclusive to the R biotype, suggesting a role in herbicide resistance. These findings underscore the importance of niche-specific microbial functions in maintaining plant health and soil ecosystem dynamics, emphasizing the need for further metagenomic or transcriptomic validation.

## Data Availability

Raw sequence data were deposited into the NCBI Sequence Read Archive (SRA) under accession number PRJNA1002228. The data that support the findings of this study are available in the supplementary material of this article.

## Supplementary Materials

The following supporting information can be downloaded at: https://www.mdpi.com/article/10.3390/plants14060876/s1, Figure S1: Bacterial Diversity and Composition; Figure S2: Taxonomic Composition Interactive Based on Krona; Figure S3: Specaccum Species Accumulation Curve, Bar Charts of ASV/OTU Abundance in Different Regions of the Petal Diagram; Figure S4: OPLS-DA Discriminant Analysis: Species Loading Plot and Sample Ordination Plot; Figure S5: Network Diagram of Dominant Species Annotated at the Phylum Level Nodes represent ASVs or OTUs in the samples, Zi-Pi Scatter Plot; Figure S6: Predicted abundance of KEGG secondary functional pathways; Figure S7: Venn diagram of fungal endophytes community in S and R populations at diverse taxonomic, In the Circos sample and species diagram, the small semicircle (left half circle) represents the species composition in the sample; Table S1: Primers used in this study; Table S2. Statistics of microbial taxa at different levels statistics of microbial taxa at different levels; Table S3. Sample information statistics.
