# Supplementary material for: Niches and Genotypes Determine the Diversity and Composition of Microbiomes After Herbicide Treatment in Beckmannia syzigachne
Source: Plants (Basel). 2025 Mar 11;14(6):876. doi: 10.3390/plants14060876 (PMC11946788; doi:10.3390/plants14060876)
Supplement: Supplementary file 1 [file plants-14-00876-s001.zip › plants-3526492-supplementary.pdf]

Supplementary material

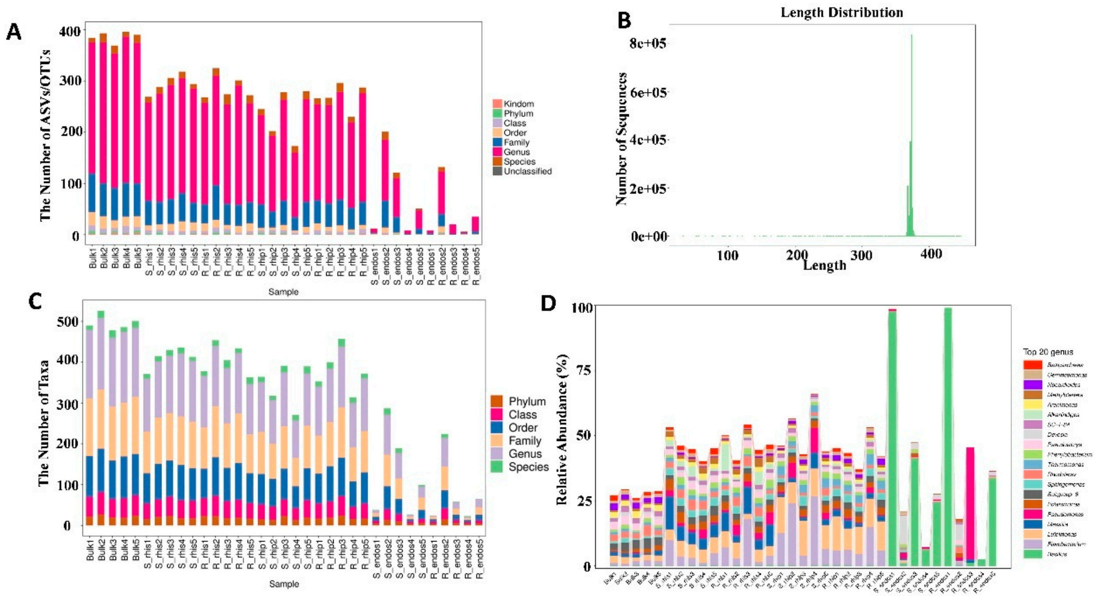

Figure S1. Bacterial Diversity and Composition. **A.** Taxonomic Annotation Results Statistics: The horizontal axis is arranged according to sample names, and the vertical axis represents the number of ASVs/OTUs in each sample that can be classified at the phylum, class, order, family, genus, and species levels. **B.** Distribution of Major Sequenced Read Lengths: This panel shows the distribution of the lengths of the primary sequences obtained from sequencing. **C.** Statistics of Microbial Taxa at Different Levels: This panel presents the count of microbial taxa at various taxonomic levels, such as phylum, class, order, family, genus, and species. **D.** Bar Chart of Species Composition at the Genus Level: This panel displays the species composition at the genus level, with the relative abundance of different genera shown as a bar chart.

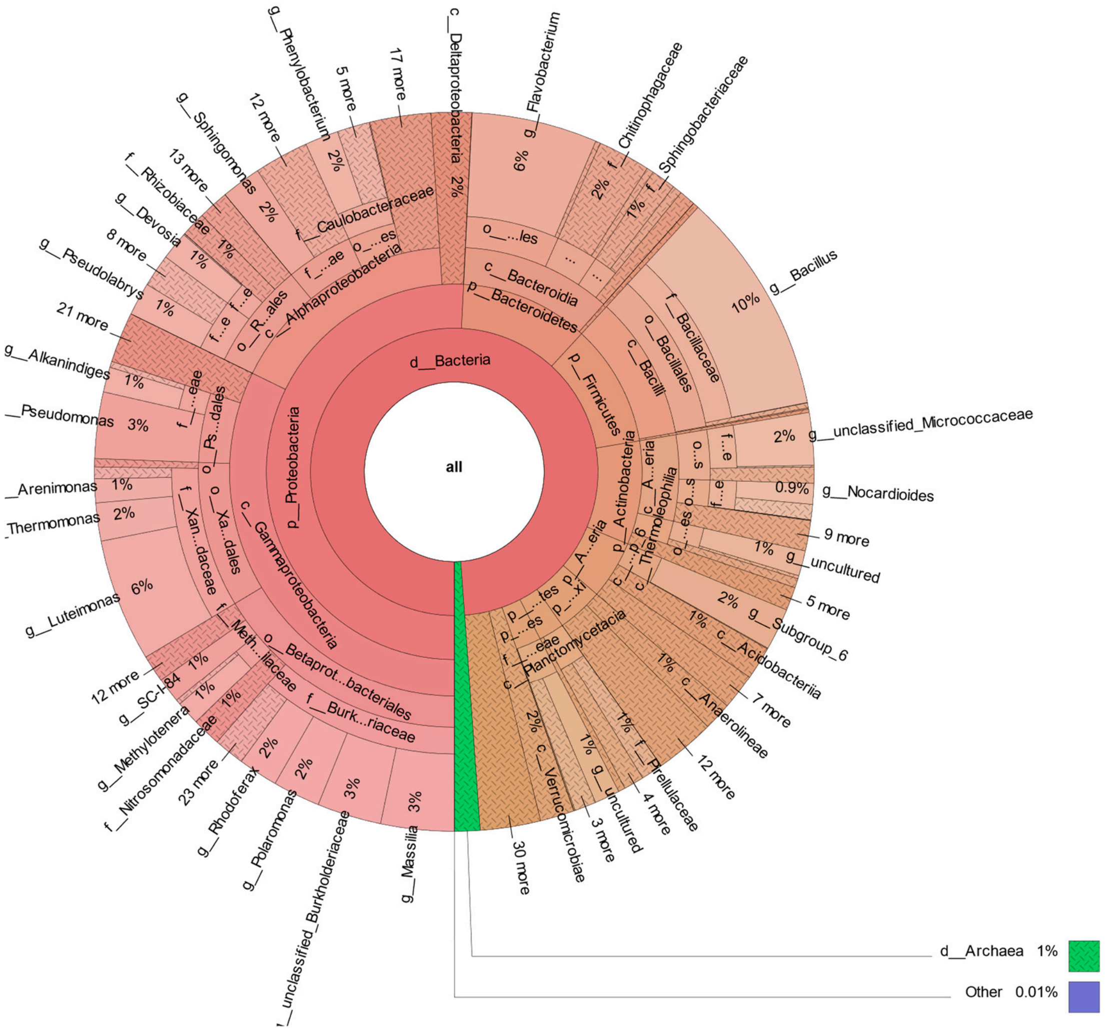

Figure S2. Taxonomic Composition Interactive Based on Krona. This figure utilizes Krona to present the taxonomic composition interactively: Each circle represents a taxonomic level from innermost to outermost: phylum, class, order, family, and genus. The size of each sector reflects the relative abundance of different taxonomic units, with specific values provided. Taxonomic units are differentiated by different colors at each taxonomic level.

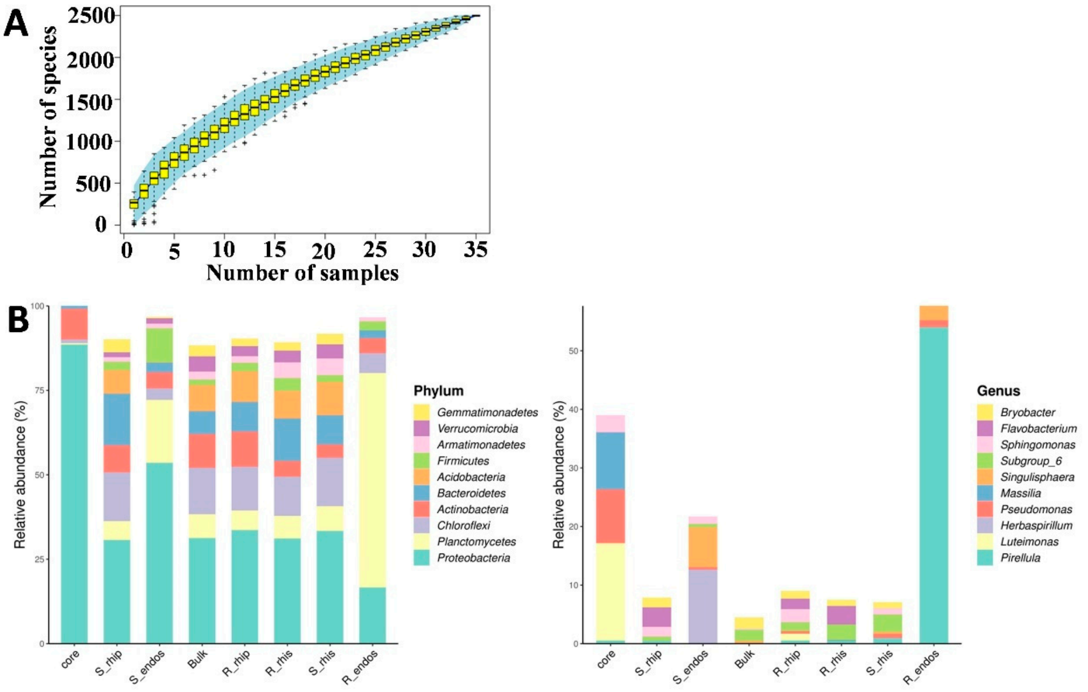

Figure S3. A. Specaccum Species Accumulation Curve. X-axis: Sample size. Y-axis: Number of observed species (ASV/OTU). Blue shading indicates the confidence interval of the curve. **B.** Bar Charts of ASV/OTU Abundance in Different Regions of the Petal Diagram, Left Chart: Abundance of sequences belonging to different phyla corresponding to ASV/OTU sets in different regions of the petal diagram. Right Chart: Abundance of sequences belonging to different genera corresponding to ASV/OTU sets in different regions of the petal diagram. X-axis: ASV/OTU sets corresponding to different regions of the petal diagram. Y-axis: Percentage of sequence abundance. Different taxonomic units are represented by different colors.

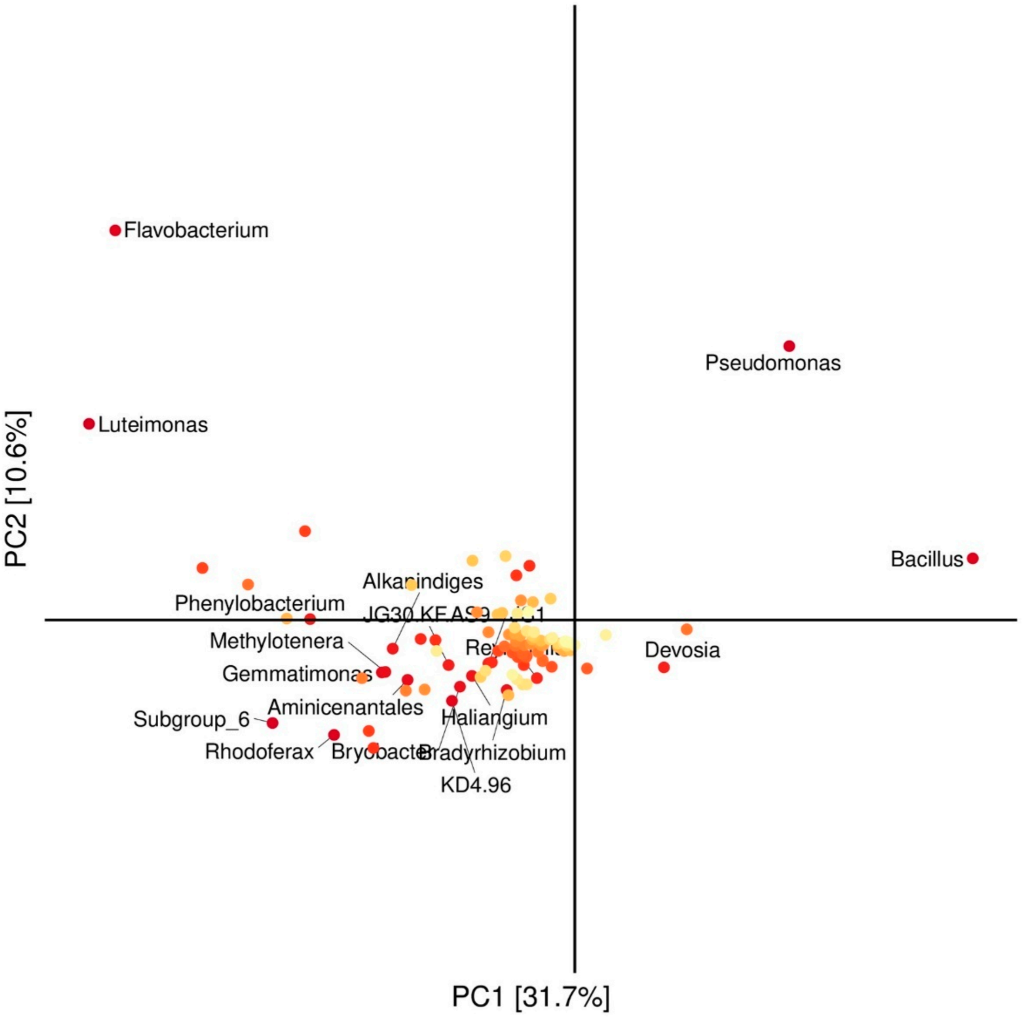

Figure S4. OPLS-DA Discriminant Analysis: Species Loading Plot and Sample Ordination Plot. Left Panel: Species Loading Plot**.** Each point represents a species (typically at the genus level). Horizontal and vertical axes represent the Variable Importance in Projection (VIP) values of the species, indicating their contribution to the differences between samples along these two dimensions. Percentages in parentheses on each axis denote the proportion of inter-group abundance differences on that dimension relative to the total inter-group difference. The physical unit length ratio on both axes corresponds to the ratio of their explanatory power, ensuring that the contribution of species to the inter-group compositional differences is proportional to their distance from the origin. Colors range from yellow to red, indicating increasing VIP values. Right Panel: Sample Ordination Plot**.** Each point represents a sample, colored differently to distinguish between different samples or groups.

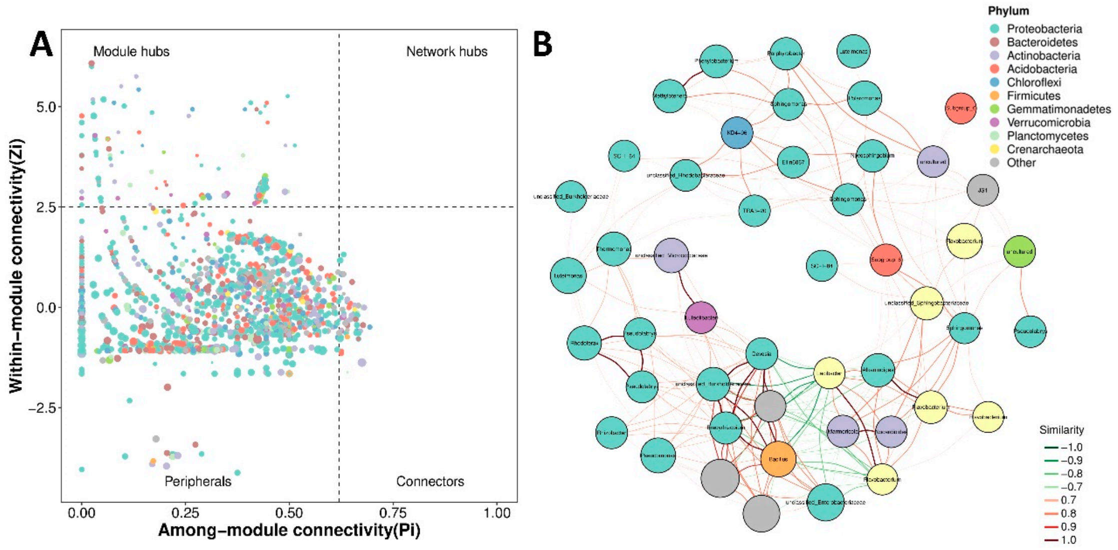

Figure S5. A. Network Diagram of Dominant Species Annotated at the Phylum Level Nodes represent ASVs or OTUs in the samples, with node size proportional to their abundance (measured in log2(CPM/n)). Only the top 50 ASVs/OTUs by average abundance across samples are displayed. Nodes are colored according to their phylum-level classification. Edges between nodes indicate correlations: red lines denote positive correlations, and green lines denote negative correlations. **B.** Zi-Pi Scatter Plot. Utilizing Zi and Pi values, nodes (ASVs/OTUs) in the network are categorized into four groups: peripherals, connectors, module hubs, and network hubs.

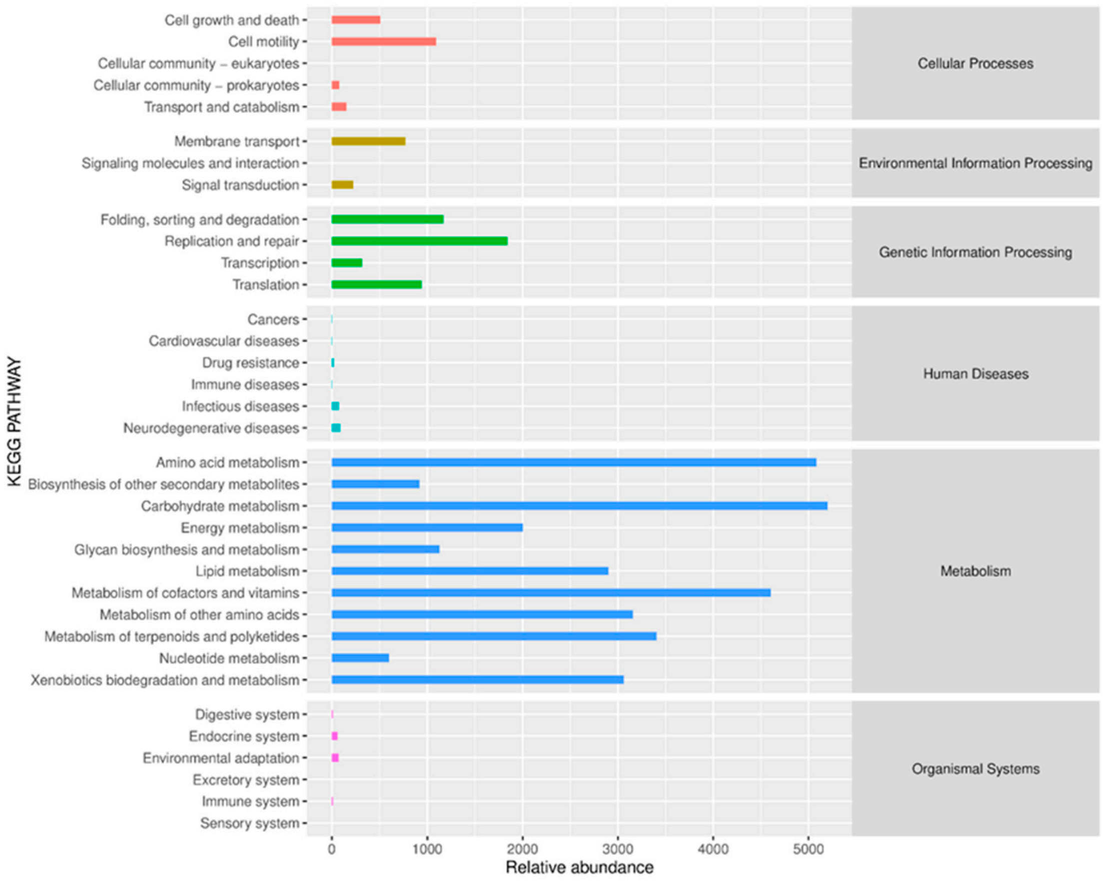

Figure S6. Predicted abundance of KEGG secondary functional pathways: The horizontal axis depicts the abundance of functional pathways (expressed in counts per million KO). The vertical axis displays the functional pathways at the second classification level of KEGG. The far-right column indicates the corresponding first-level pathways associated with these secondary pathways.

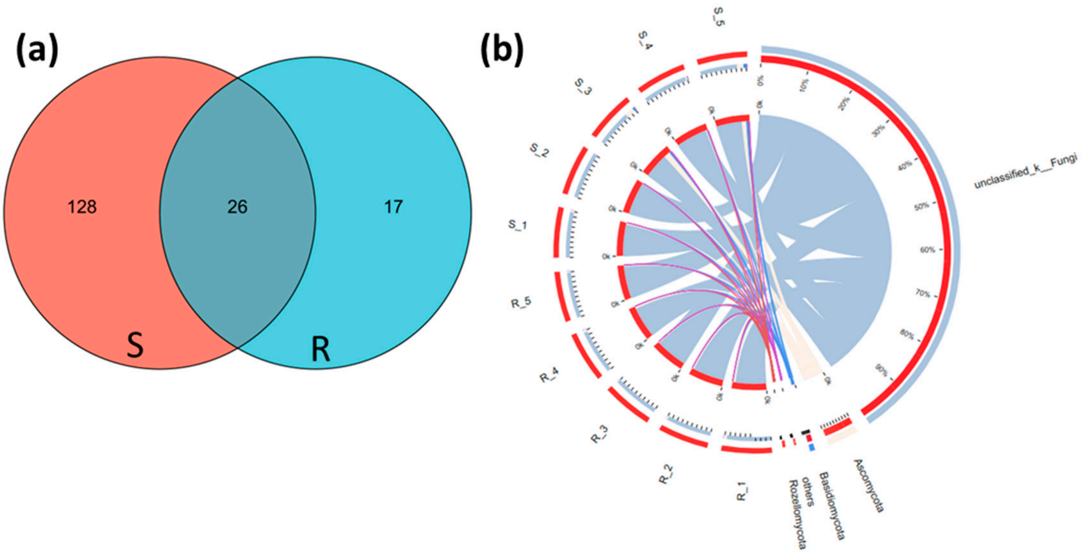

Figure S7. A. Venn diagram of fungal endophytes community in S and R populations at diverse taxnomic; **B.** In the Circos sample and species diagram, the small semicircle (left half circle) represents the species composition in the sample, the color of the outer band represents the group from which the color of the inner band represents the species, and the length represents the relative abundance of the species in the corresponding sample. The large semicircle (right half circle) represents the distribution ratio of species in different samples at the taxonomic level. The outer ribbon represents the species, the inner ribbon colors represent different groups, and the length represents the distribution ratio of the sample in a certain species.

Tables

Table S1. Primers used in this study

Primers	Sequence (5'-3')
338F	ACTCCTACGGGAGGCAGCA
806R	GGACTACHVGGGTWTCTAAT
ITS1F	CTTGGTCATTTAGAGGAAGTAA
ITS2R	GCTGCGTTCTTCATCGATGC

Table S2. Statistics of microbial taxa at different levels statistics of microbial taxa at different levels

Sample	Phylum	Class	Order	Family	Genus	Species
Bulk1	22	48	100	141	168	11
Bulk2	27	54	107	145	175	18
Bulk3	20	44	95	132	169	18
Bulk4	19	47	103	132	173	12
Bulk5	24	49	102	140	169	17
R_endos1	4	5	6	6	3	0
R_endos2	15	26	45	58	70	10
R_endos3	6	7	13	15	16	1
R_endos4	4	4	5	5	3	2
R_endos5	5	7	14	18	20	1
R_rhip1	17	36	74	93	120	13
R_rhip2	19	39	87	108	131	16
R_rhip3	24	47	95	123	149	19
R_rhip4	16	29	63	83	109	14
R_rhip5	17	36	77	101	129	12
R_rhis1	23	43	73	101	126	12
R_rhis2	23	49	95	125	147	15
R_rhis3	18	41	82	109	135	20
R_rhis4	19	43	91	121	148	12
R_rhis5	17	37	74	95	123	17
S_endos1	5	6	10	9	7	1
S_endos2	14	25	57	77	98	16
S_endos3	10	17	38	50	62	12
S_endos4	4	5	6	6	5	1
S_endos5	6	9	23	30	27	4
S_rhip1	15	36	75	103	122	13
S_rhip2	14	32	68	86	107	11
S_rhip3	23	40	76	104	132	16
S_rhip4	14	28	54	69	92	14
S_rhip5	19	42	79	104	128	18
S_rhis1	16	37	75	102	129	12
S_rhis2	21	42	88	113	138	13
S_rhis3	23	47	89	116	140	15
S_rhis4	18	43	87	119	154	15
S_rhis5	18	42	80	114	149	10

Table S3. Sample information statistics

Sample	reads	bases	avg	min	max
S_1	48559	14441420	297.399452	195	361
S_2	43322	12947780	298.87309	143	306
S_3	48489	13863331	285.906721	161	510
S_4	41082	12198269	296.924906	142	437
S_5	44423	12879091	289.919434	150	491
R_1	43330	13090608	302.114193	158	438
R_2	35882	10659303	297.065465	159	437
R_3	39098	11656380	298.132385	195	437
R_4	41051	12286852	299.307008	142	524
R_5	47744	14239612	298.249246	143	437

The first column is the sample name, and the second column is the relevant information of the sample, which is sequence number, base number, average length, shortest sequence length, and longest sequence length.
